# Majorization involving the cyclic moving average

**DOI:** 10.1186/s13660-018-1737-4

**Published:** 2018-06-28

**Authors:** Tao Zhang, Huan-Nan Shi, Bo-Yan Xi, Alatancang Chen

**Affiliations:** 1 School of Mathematical Sciences, Inner Mongolia University, Hohhot, China; 2 Teacher’s College, Beijing Union University, Beijing, China; 3 College of Mathematics, Inner Mongolia University for Nationalities, Tongliao, China

**Keywords:** 26D15, 26E60, 26A51, Inequality, Cyclic moving average

## Abstract

We solve an open problem on some majorization inequalities involving the cyclic moving average.

## Introduction

We first recall two definitions.

### Definition 1.1

([1])

For fixed $n\geq 2$, let $x=(x_{1},x_{2},\ldots ,x_{n})$ and $y=(y_{1},y_{2},\ldots ,y_{n})$ be two *n*-tuples of real numbers.

(i) *x* is said to be majorized by *y* (in symbols, $x \prec y$) if
  $$ \sum_{i = 1}^{k} x_{[i]} \le \sum _{i = 1}^{k} y_{[i]}\quad\mbox{for }k = 1,2,\ldots ,n -1,\quad \mbox{and} \quad \sum_{i = 1}^{n} x_{i} = \sum_{i = 1}^{n} y_{i}, $$
  where $x_{[1]}\ge \cdots \ge x_{[n]}$ and $y_{[1]}\ge \cdots \ge y _{[n]}$ are rearrangements of *x* and *y* in descending order.
(ii) Let $\Omega \subset \mathbb{R}^{n}$. A function $\varphi :\Omega \rightarrow \mathbb{R}$ is said to be a Schur-convex function (shortly, an *S*-convex function) if
  $$ x \prec y \Rightarrow \varphi (x) \leq \varphi (y). $$

For example:

$$\begin{aligned}& a^{(2)}= \biggl( \frac{a_{1}+a_{2}}{2},\frac{a_{2}+a_{3}}{2},\ldots , \frac{a _{n}+a_{1}}{2} \biggr) , \\& a^{(3)}= \biggl( \frac{a_{1}+a_{2}+a_{3}}{3},\frac{a_{2}+a_{3}+a_{4}}{3}, \ldots , \frac{a_{n}+a_{1}+a_{2}}{3} \biggr) . \end{aligned}$$

In 2006, I. Olkin, one of the authors of the book [1], wrote a letter to K. Z. Guan, referring to the following interesting question: is it true that

1 $$ a^{(k+1)}\prec a^{(k)},\quad 1\leq k\leq n-1? $$

However, a proof for $a^{(k+1)}\prec a^{(k)}$ remains elusive (see [1], p. 63).

In 2010, Shi [2] proved that (1) holds when $n= 4$, $k= 2 $ and $n=5$, $k=3 $. In this paper, we prove that (1) holds for any $n\geq 2$ and $1\leq k\leq n-1$.

For any $1\leq k\leq n$, let

$$ a^{(k)}_{[1]}\geq a^{(k)}_{[2]}\geq \cdots \geq a^{(k)}_{[n]} $$

be the ordered component of the sequence $a^{(k)}_{1},a^{(k)}_{2}, \ldots ,a^{(k)}_{n}$. We denote

$$ S_{h}^{(k)}=\sum _{i=1}^{h}a^{(k)}_{[i]}, \quad 1\leq h\leq n. $$

## Lemmas and corollaries

For proving our main results, we need the following lemmas.

### Lemma 2.1

*Let* $n\geq 2$ *and* $1\le k\le n-1$. *Then*

2 $$ \textstyle\begin{cases} a^{(k)}_{1}\geq a^{(k)}_{2}\geq \cdots \geq a^{(k)}_{n-k+1}, \\ a^{(k)}_{n}\geq a^{(k)}_{n-1}\geq \cdots \geq a^{(k)}_{n-k+1}, \\ a^{(k)}_{1}\geq a^{(k)}_{n}. \end{cases} $$

### Proof

For any $1\le k\le n-1$ and $1\le i\le n$, we have

$$ k\bigl(a_{i}^{(k)}-a_{i+1}^{(k)}\bigr)= ( a_{i}+a_{i+1}+\cdots +a_{i+k-1} ) - ( a_{i+1}+a_{i+2}+\cdots +a_{i+k} ) =a_{i}-a_{i+k}. $$

Note that $a_{1}\geq a_{2}\geq \cdots \geq a_{n}$, so we can induce that

1. if $i+k\leq n$, that is, $1\leq i\leq n-k$, then $a_{i}\geq a_{i+k}$. It follows that $a_{i}^{(k)}\geq a_{i+1}^{(k)}$.
2. if $i+k> n$, that is, $n-k< i\leq n$, then $a_{i+k}=a_{n+(i+k-n)}=a _{i+k-n}$. Since $1\le k\le n-1$, we have $n\geq i> i+k-n$. So $a_{i}\geq a_{i+k-n}=a_{i+k}$. It follows that $a_{i}^{(k)}\geq a_{i+1} ^{(k)}$.
3. $k(a_{1}^{(k)}-a_{n}^{(k)})= ( a_{1}+a_{2}+\cdots +a_{k} ) - ( a_{n}+a_{1}+\cdots +a_{k-1} ) =a_{k}-a_{n}\geq 0$. Therefore (2) holds.

 □

From the proof of Lemma 2.1 it is easy to deduce the following:

### Corollary 2.2

*Let* $n\geq 2$, $1\leq k\leq n-1$, *and let* $\sum_{i=0}^{-1}a^{(k)}_{n-i}=0$. *For any* $1\leq h\leq n-1$, *there exist* $1\leq h_{1}\leq n-k$ *and* $-1\leq h_{2}\leq k-2$ *such that* $h=h_{1}+h _{2}+1$ *and*

$$ S_{h}^{(k)}= \sum_{i=1}^{h_{1}}a^{(k)}_{i}+ \sum_{i=0}^{h_{2}}a^{(k)} _{n-i}. $$

### Lemma 2.3

*Let* $n\geq 4$, $2\leq k\leq n-2$, *and* $0\leq r\leq k-2$.

(i) *If*
  3 $$ a^{(k)}_{2}\leq a^{(k)}_{n-r} \leq a^{(k)}_{1}, $$
  *then*
  $$ a^{(k+1)}_{2}\leq a^{(k+1)}_{n-r} \leq a^{(k+1)}_{1}. $$
(ii) *If*
  4 $$ a^{(k)}_{n-k+1}\leq a^{(k)}_{n-r} \leq a^{(k)}_{n-k}, $$
  *then*
  $$ a^{(k+1)}_{n-k}\leq a^{(k+1)}_{n-r-1} \leq a^{(k+1)}_{n-k-1}. $$
(iii) *For* $2\leq m\leq n-k-1$, *if*
  5 $$ a^{(k)}_{m+1}\leq a^{(k)}_{n-r} \leq a^{(k)}_{m}, $$
  *then*
  6 $$ a^{(k+1)}_{m+1}\leq a^{(k+1)}_{n-r}, \qquad a^{(k+1)}_{n-r-1}\leq a^{(k+1)}_{m-1}. $$
(iv) *If*
  7 $$ a^{(k)}_{n}\leq a^{(k)}_{n-k} , $$
  *then*
  $$ a^{(k+1)}_{n-1}\leq a^{(k+1)}_{n-k-1} . $$
(v) *For* $2\leq m\leq n-k-1$, *if*
  8 $$ a^{(k)}_{n}\leq a^{(k)}_{m} , $$
  *then*
  $$ a^{(k+1)}_{n-1}\leq a^{(k+1)}_{m} . $$
(vi) *For* $2\leq m\leq n-k$, *if*
  9 $$ a^{(k)}_{n-r-1}\leq a^{(k)}_{m} \leq a^{(k)}_{n-r}, $$
  *then*
  $$ a^{(k+1)}_{n-r-2}\leq a^{(k+1)}_{m-1}, \qquad a^{(k+1)}_{m}\leq a^{(k+1)}_{n-r}. $$

### Proof

(i) By Lemma 2.1 we have
  $$ \textstyle\begin{cases} a^{(k+1)}_{1}\geq a^{(k+1)}_{2}\geq \cdots \geq a^{(k+1)}_{n-k}, \\ a^{(k+1)}_{n}\geq a^{(k+1)}_{n-1}\geq \cdots \geq a^{(k+1)}_{n-k}, \\ a^{(k+1)}_{1}\geq a^{(k+1)}_{n}. \end{cases} $$
  It follows that
  $$ a^{(k+1)}_{1}=\max \bigl\{ a^{(k+1)}_{1}, a^{(k+1)}_{2},\ldots , a^{(k+1)} _{n}\bigr\} . $$
  Thus we have
  $$ a^{(k+1)}_{n-r}\leq a^{(k+1)}_{1}. $$
  By the left inequality of (3) we have
  $$ a_{2}+ \cdots + a_{k+1}\leq a_{n-r}+ \cdots + a_{n-r+k-1}. $$
  Note that $a_{k+2}\leq a_{k-r}=a_{n+k-r}$, so we have
  $$ a_{2}+ \cdots + a_{k+1}+ a_{k+2}\leq a_{n-r}+\cdots + a_{n-r+k-1}+ a _{n+k-r}. $$
  Therefore
  $$ a^{(k+1)}_{2}\leq a^{(k+1)}_{n-r}. $$
(ii) By Lemma 2.1 we have
  $$ a^{(k+1)}_{n-k}\leq a^{(k+1)}_{n-r-1}. $$
  By the right inequality of (4) we get
  $$ a_{n-r} +\cdots + a_{n-r+k-1}\leq a_{n-k} +\cdots +a_{n-1}. $$
  Note that $a_{n-r-1}\leq a_{n-k-1}$, so we have
  $$ a_{n-r} +\cdots + a_{n-r+k-1} + a_{n-r-1}\leq a_{n-k} +\cdots +a_{n-1}+ a_{n-k-1}. $$
  This means that
  $$ a^{(k+1)}_{n-r-1}\leq a^{(k+1)}_{n-k-1}. $$
(iii) By (5) we get
  $$ a_{m+1}+ \cdots + a_{m+k}\leq a_{n-r} + \cdots + a_{n-r+k-1} \leq a _{m}+ \cdots +a_{m+k-1}. $$
  Note that $n\geq m+k+1\geq k-r \geq 1$ and $n\geq n-r-1\geq m-1 \geq 1$, so we have
  $$ a_{m+k+1}\leq a_{n-r+k}=a_{k-r},\qquad a_{n-r-1} \leq a_{m-1}. $$
  It follows that
  $$ a_{m+1}+ \cdots + a_{m+k} + a_{m+k+1}\leq a_{n-r} + \cdots + a_{n-r+k-1} + a_{n-r+k} $$
  and
  $$ a_{n-r-1} + a_{n-r} + \cdots + a_{n-r+k-1} \leq a_{m-1} +a_{m}+ \cdots +a_{m+k-1}. $$
  Therefore (6) holds.
(iv) By (7) we get
  $$ a_{n} + \cdots + a_{n+k-1}\leq a_{n-k} + \cdots + a_{n-1}. $$
  Since $a_{n-1}\leq a_{n-k-1}$, we have
  $$ a_{n} + \cdots + a_{n+k-1} + a_{n-1}\leq a_{n-k} + \cdots + a_{n-1} + a_{n-k-1}. $$
  It follows that
  $$ a^{(k+1)}_{n-1}\leq a^{(k+1)}_{n-k-1}. $$
(v) By (8) we have
  $$ a_{n} + \cdots + a_{n+k-1}\leq a_{m} +\cdots + a_{m+k-1}. $$
  Note that $n-1\geq m+k$ and $a_{n-1}\leq a_{m+k}$, so we have
  $$ a_{n} + \cdots + a_{n+k-1}+ a_{n-1}\leq a_{m} +\cdots + a_{m+k-1}+ a _{m+k}. $$
  This means that
  $$ a^{(k+1)}_{n-1}\leq a^{(k+1)}_{m}. $$
(vi) By (9) we get
  $$ a_{n-r-1}+\cdots + a_{n-r+k-2}\leq a_{m} +\cdots + a_{m+k-1}\leq a _{n-r}+\cdots + a_{n-r+k-1}. $$
  Note that $0\leq r\leq k-2$ and $2\leq m\leq n-k$, so we have
  $$ n\geq n-r-2\geq n-k\geq m\geq m-1\geq 1. $$
  It follows that
  $$ a_{n-r-2}\leq a_{m-1}. $$
  So we get
  $$ a_{n-r-2} + a_{n-r-1}+\cdots + a_{n-r+k-2}\leq a_{m-1} + a_{m} + \cdots + a_{m+k-1}. $$
  Therefore
  $$ a^{(k+1)}_{n-r-2}\leq a^{(k+1)}_{m-1}. $$
  Note that $m\leq n-k$, so we have
  $$ k-r\leq m+k\leq n,\qquad a_{m+k}\leq a_{k-r}= a_{n+k-r}. $$
  It follows that
  $$ a_{m} +\cdots + a_{m+k-1} + a_{m+k}\leq a_{n-r}+\cdots + a_{n-r+k-1} + a_{n+k-r}. $$
  Therefore
  $$ a^{(k+1)}_{m}\leq a^{(k+1)}_{n-r}. $$

 □

### Lemma 2.4

*Let* $n\geq 4$, $2\leq k\leq n-1$, $1\leq m\leq n-k$, *and* $0\leq r \leq k-2$.

(i) *If* $a^{(k)}_{m+1}\leq a^{(k)}_{n-r}\leq a^{(k)}_{m}$, *then*
  10 $$ S_{m+r+1}^{(k)}= \sum _{i=1}^{m}a^{(k)}_{i}+\sum _{i=0}^{r}a^{(k)}_{n-i}. $$
(ii) *If* $a^{(k)}_{n}\leq a^{(k)}_{m} $, *then*
  $$ S_{m}^{(k)}= \sum_{i=1}^{m}a^{(k)}_{i}. $$
(iii) *For* $2\leq m\leq n-k$, *if* $a^{(k)}_{n-r-1}\leq a^{(k)} _{m}\leq a^{(k)}_{n-r}$, *then*
  $$ S_{m+r+1}^{(k)}= \sum_{i=1}^{m}a^{(k)}_{i}+ \sum_{i=0}^{r}a^{(k)}_{n-i}. $$

### Proof

We only prove (i). Using a similar method, we can obtain (ii) and (iii).

By Lemma 2.1 we have

11 $$ \textstyle\begin{cases} a_{1}^{(k)}\geq a_{2}^{(k)}\geq \cdots \geq a^{(k)}_{m} \geq a^{(k)} _{n-r}\geq a^{(k)}_{m+1}, \\ a^{(k)}_{n}\geq a^{(k)}_{n-1}\geq \cdots \geq a^{(k)}_{n-r+1}\geq a ^{(k)}_{n-r}\geq a^{(k)}_{m+1}. \end{cases} $$

By Corollary 2.2 we let

12 $$ S_{m+r+1}^{(k)}=\sum_{i=1}^{h_{1}}a^{(k)}_{i}+ \sum_{i=0}^{h_{2}}a^{(k)} _{n-i}, $$

where $1\leq h_{1}\leq n-k+1$, $-1\leq h_{2}\leq k-2$, and $\sum_{i=0} ^{-1}a^{(k)}_{n-i}=0$. It is clear that

13 $$ m+r+1= h_{1}+h_{2}+1. $$

Next, we prove that $h_{1}=m$ and $h_{2}=r$.

1. If $h_{1}\geq m+1$, which means that the right-hand side of (12) includes $a^{(k)}_{m+1}$, then by (11) the right-hand side of (12) should include $a^{(k)}_{1},a^{(k)} _{2},\ldots ,a^{(k)}_{m+1}$ and $a^{(k)}_{n-r}, a^{(k)}_{n-r+1}, \ldots , a^{(k)}_{n}$, so we have
  $$ h_{1}+h_{2}+1\geq m+r+2>m+r+1. $$
  This is a contradiction with (13).
2. If $h_{1}\leq m-1$, then by (13) we have $k-2\geq h_{2} \geq r+1$. Together with (11), we get
  $$ a^{(k)}_{n-h_{2}}\leq a^{(k)}_{n-r-1}\leq a^{(k)}_{n-r}\leq a^{(k)} _{m}. $$
  So the right-hand side of (12) must include $a_{m}^{(k)}$, which means that $h_{1}\geq m$. This is a contradiction with $h_{1}\leq m-1$.

Therefore $h_{1}=m$ and $h_{2}=r$. So (10) holds. □

### Corollary 2.5

*Let* $n\geq 4$, $2\leq k\leq n-2$, $1\leq m\leq n-k$, *and* $0\leq r \leq k-2$, *and let*

14 $$ a^{(k)}_{m+1}\leq a^{(k)}_{n-r} \leq a^{(k)}_{m}. $$

(i) *For* $m=1$, *we have*
  15 $$ S_{r+2}^{(k+1)}=a^{(k+1)}_{1}+ \sum_{i=0}^{r}a^{(k+1)}_{n-i}. $$
(ii) *For* $m=n-k$, *we have*
  16 $$ S_{n-k+r+1}^{(k+1)}= \sum _{i=1}^{n-k-1}a^{(k+1)}_{i}+ \sum _{i=0}^{r+1}a^{(k+1)}_{n-i}. $$
(iii) *For* $2\leq k\leq n-3$ *and* $2\leq m\leq n-k-1$, $S_{m+r+1}^{(k+1)}$ *must be one of the following two cases*:
  $$ S_{m+r+1}^{(k+1)}=\sum_{i=1}^{m}a^{(k+1)}_{i}+ \sum_{i=0}^{r}a^{(k+1)} _{n-i}, $$
  *or*
  $$ S_{m+r+1}^{(k+1)}=\sum_{i=1}^{m-1}a^{(k+1)}_{i}+ \sum_{i=0}^{r+1}a^{(k+1)} _{n-i}. $$

### Proof

(i) If $m=1$, by (14) we have
  $$ a_{2}^{(k)}\leq a^{(k)}_{n-r}\leq a_{1}^{(k)}. $$
  By Lemma 2.3(i) we have
  $$ a^{(k+1)}_{2}\leq a^{(k+1)}_{n-r}\leq a^{(k+1)}_{1}, $$
  and then by Lemma 2.4(i) we can induce that (15) holds.
(ii) If $m=n-k$, then by (14) we have
  $$ a^{(k)}_{n-k+1}\leq a^{(k)}_{n-r}\leq a^{(k)}_{n-k}. $$
  By Lemma 2.3(ii) we have
  $$ a^{(k+1)}_{n-k}\leq a^{(k+1)}_{n-r-1}\leq a^{(k+1)}_{n-k-1}, $$
  and then by Lemma 2.4(i) we can induce that (16) holds.
(iii) By Corollary 2.2 we let
  17 $$ S_{m+r+1}^{(k+1)}=\sum_{i=1}^{p}a^{(k+1)}_{i}+ \sum_{i=0}^{q}a^{(k+1)} _{n-i}, $$
  where $1\leq p\leq n-k$, $-1\leq q\leq k-2$, and $\sum_{i=0}^{-1}a^{(k)} _{n-i}=0$. Then we have
  18 $$ p+q+1= m+r+1. $$

Next, we prove that $p=m$ or $p=m-1$.

1. If $p\geq m+1$, then by Lemma (2.3)(iii) we have $a^{(k+1)}_{m+1}\leq a^{(k+1)}_{n-r}$. So we get
  $$ a^{(k+1)}_{p}\leq a^{(k+1)}_{m+1}\leq a^{(k+1)}_{n-r}. $$
  Thus the right-hand side of (17) includes $a^{(k+1)}_{n-r}$, which means that $q \geq r$. Therefore
  $$ p+q+1\geq m+r+2> m+r+1. $$
  This is a contradiction with (18).
2. If $1\leq p\leq m-2$, then by (18) we have $n-q\leq n-r-2$. By Lemma 2.3(iii) we get $a^{(k+1)}_{n-r-1}\leq a^{(k+1)} _{m-1}$. It follows that
  $$ a^{(k+1)}_{n-q}\leq a^{(k+1)}_{n-r-2}\leq a^{(k+1)}_{n-r-1}\leq a^{(k+1)} _{m-1}. $$
  So the right-hand side of (17) must include $a^{(k+1)}_{m-1}$. Therefore
  $$ p \geq m-1. $$
  This is a contradiction with $1\leq p\leq m-2$.

Thus $p=m$ or $p=m-1$. □

In a similar way as in Corollary 2.5, we can prove the following corollaries.

### Corollary 2.6

*Let* $n\geq 4$, $2\leq k\leq n-2$, $2\leq m\leq n-k$, *and* $0\leq r \leq k-2$.

(i) *If* $a^{(k)}_{n}\leq a^{(k)}_{m} $, *then* $S_{m}^{(k+1)}$ *must be one of the following two cases*:
  $$ S_{m}^{(k+1)}= \sum_{i=1}^{m}a^{(k+1)}_{i} $$
  *or*
  $$ S_{m}^{(k+1)}=\sum_{i=1}^{m-1}a^{(k+1)}_{i}+a^{(k+1)}_{n}. $$
(ii) *If* $a^{(k)}_{n-r-1}\leq a^{(k)}_{m}\leq a^{(k)}_{n-r}$, *then* $S_{m+r+1}^{(k+1)}$ *must be one of the following two cases*:
  $$ S_{m+r+1}^{(k+1)}=\sum_{i=1}^{m}a^{(k+1)}_{i}+ \sum_{i=0}^{r}a^{(k+1)} _{n-i} $$
  *or*
  $$ S_{m+r+1}^{(k+1)}=\sum_{i=1}^{m-1}a^{(k+1)}_{i}+ \sum_{i=0}^{r+1}a^{(k+1)} _{n-i}. $$

### Corollary 2.7

*Let* $n\geq 4$, $2\leq k\leq n-2$, $1\leq m\leq n-k$, *and* $-1\leq r \leq k-2$, *and let* $\sum_{i=0}^{-1}a^{(k)}_{n-i}=0$. *If*

$$ S_{m+r+1}^{(k)}= \sum _{i=1}^{m}a^{(k)}_{i}+\sum _{i=0}^{r}a^{(k)}_{n-i}, $$

*then we have*:

(i) *if* $m=1$, *then*
  $$ S_{r+2}^{(k+1)}=a^{(k+1)}_{1}+ \sum_{i=0}^{r}a^{(k+1)}_{n-i}; $$
(ii) *if* $2\leq m\leq n-k$, *then* $S_{m+r+1}^{(k+1)}$ *must be one of the following two cases*:
  $$ S_{m+r+1}^{(k+1)}=\sum_{i=1}^{m}a^{(k+1)}_{i}+ \sum_{i=0}^{r}a^{(k+1)} _{n-i} $$
  *or*
  $$ S_{m+r+1}^{(k+1)}=\sum_{i=1}^{m-1}a^{(k+1)}_{i}+ \sum_{i=0}^{r+1}a^{(k+1)} _{n-i}. $$

### Lemma 2.8

*Let* $n\geq 4$, $2\leq k\leq n-2$, $1\leq m\leq n-k$, *and* $-1\leq r \leq k-2$, *and let* $\sum_{i=0}^{-1}a^{(k)}_{n-i}=0$. *If*

19 $$ \textstyle\begin{cases} S_{m+r+1}^{(k)}= \sum_{i=1}^{m}a^{(k)}_{i}+\sum_{i=0}^{r}a^{(k)}_{n-i}, \\ S_{m+r+1}^{(k+1)}= \sum_{i=1}^{m}a^{(k+1)}_{i}+\sum_{i=0}^{r}a^{(k+1)}_{n-i}, \end{cases} $$

*then*

20 $$ S_{m+r+1}^{(k)}\geq S_{m+r+1}^{(k+1)}. $$

### Proof

By a simple calculation we obtain

$$ S_{m+r+1}^{(k)}-S_{m+r+1}^{(k+1)}= \frac{1}{k+1} \Biggl( S_{m+r+1}^{(k)}- \sum _{i=k-r}^{k+m}a_{i} \Biggr) . $$

By (19) we have

$$ a^{(k)}_{m+1}\leq a^{(k)}_{n-r}. $$

It follows that

$$ a_{m+1}+a_{m+2}+\cdots +a_{k+m}\leq a_{n-r}+a_{n-r+1}+\cdots +a_{n-r+k-1}. $$

So we have

$$ a_{k-r}+a_{k-r+1}+\cdots +a_{k+m}\leq a_{n-r}+a_{n-r+1}+\cdots +a_{n+m}. $$

Note that

$$ \textstyle\begin{cases} a_{n-r}+a_{n-r+1}+\cdots +a_{n+m}\leq a_{n-r+j}+a_{n-r+1+j}+\cdots +a _{n+m+j},& 0\leq j\leq r, \\ a_{k-r}+a_{k-r+1}+\cdots +a_{k+m}\leq a_{n-r+j}+a_{n-r+1+j}+\cdots +a _{n+m+j}, &r+1\leq j\leq k-1. \end{cases} $$

Thus we can induce that

$$ S_{m+r+1}^{(k)}= \sum _{i=1}^{m}a^{(k)}_{i}+\sum _{i=0}^{r}a^{(k)}_{n-i}= \frac{1}{k}\sum_{i=n-r}^{n+m}\sum _{j=0}^{k-1} a_{j+i} = \frac{1}{k} \sum_{j=0}^{k-1}\sum _{i=n-r}^{n+m}a_{j+i} \geq \sum _{i=k-r}^{k+m}a _{i}. $$

This means that (20) holds. □

### Lemma 2.9

*Let* $n\geq 4$, $2\leq k\leq n-2$, $2\leq m\leq n-k$, *and* $-1\leq r \leq k-2$, *and let* $\sum_{i=0}^{-1}a^{(k)}_{n-i}=0$. *If*

21 $$ \textstyle\begin{cases} S_{m+r+1}^{(k)}= \sum_{i=1}^{m}a^{(k)}_{i}+\sum_{i=0}^{r}a^{(k)}_{n-i}, \\ S_{m+r+1}^{(k+1)}= \sum_{i=1}^{m-1}a^{(k+1)}_{i}+\sum_{i=0}^{r+1}a^{(k+1)}_{n-i}, \end{cases} $$

*then*

22 $$ S_{m+r+1}^{(k)}\geq S_{m+r+1}^{(k+1)}. $$

### Proof

Note that

$$\begin{aligned} &S_{m+r+1}^{(k)}-S_{m+r+1}^{(k+1)} \\ &\quad = \frac{1}{k+1} \Biggl( \sum _{i=1}^{m-1}a^{(k)}_{i}+ \sum _{i=0}^{r}a^{(k)}_{n-i} \Biggr) +a^{(k)}_{m}-a^{(k+1)}_{n-r-1}- \frac{1}{k+1} \sum _{i=k-r}^{k+m-1}a_{i} \\ &\quad = \frac{1}{k+1} \Biggl( \sum _{i=1}^{m}a^{(k)}_{i}+ \sum _{i=0}^{r}a^{(k)}_{n-i}- \sum _{i=n-r-1}^{n+m-1}a_{i} \Biggr) \\ &\quad =\frac{1}{k+1} \Biggl( S_{m+r+1}^{(k)}-\sum _{i=n-r-1}^{n+m-1}a _{i} \Biggr) . \end{aligned}$$

By (21) we have

$$ a^{(k)}_{n-r-1}\leq a^{(k)}_{m}. $$

It follows that

$$ a_{n-r-1}+a_{n-r}+\cdots +a_{n-r+k-2}\leq a_{m}+a_{m+1}+\cdots +a_{m+k-1}. $$

So we can induce that

$$ a_{n-r-1}+a_{n-r}+\cdots +a_{n+m-1}\leq a_{k-r-1}+a_{k-r}+\cdots +a _{k+m-1}. $$

Since

$$ \textstyle\begin{cases} a_{n-r-1}+a_{n-r}+\cdots +a_{n+m-1}\leq a_{n-r+j}+a_{n-r+1+j}+\cdots +a_{n+m+j}, &0\leq j\leq r, \\ a_{k-r-1}+a_{k-r}+\cdots +a_{k+m-1}\leq a_{n-r+j}+a_{n-r+1+j}+\cdots +a_{n+m+j}, &r+1\leq j\leq k-1, \end{cases} $$

we have

$$ S_{m+r+1}^{(k)} =\frac{1}{k} \sum _{j=0}^{k-1}\sum_{i=n-r}^{n+m}a_{j+i} \geq \sum_{i=n-r-1}^{n+m-1}a_{i}. $$

This means that (22) holds. □

## Main results

We are now in a position to prove our main results (1) in two cases: $k=1$ and $2 \leq k\leq n-1$.

### Theorem 3.1

*For any* $n\geq 2$, *we have*

23 $$ a^{(2)}\prec a^{(1)}=a. $$

### Proof

It is clear that (23) holds if $n=2$. Next, let $n\geq 3$. Then we have

$$ \frac{a_{1}+a_{2}}{2}= S_{1}^{(2)}\leq S_{1}^{(1)}=a_{1}, \qquad S_{n}^{(2)}=S _{n}^{(1)}. $$

For $2\leq m\leq n-1$, we prove that $S_{m}^{(2)}\leq S_{m}^{(1)}$ in the following two cases:

(i) If $S_{m}^{(2)}= \sum_{i=1}^{m}a^{(2)}_{i}$, then
  $$ S_{m}^{(2)}-S_{m}^{(1)}= \frac{a_{m+1}-a_{1}}{2}\leq 0. $$
(ii) If $S_{m}^{(2)}= a^{(2)}_{n}+\sum_{i=1}^{m-1}a^{(2)}_{i}$, then
  $$ S_{m}^{(2)}-S_{m}^{(1)}= \frac{a_{n}-a_{m}}{2}\leq 0. $$
  So (23) holds.

 □

### Theorem 3.2

*For any* $n\geq 3$ *and* $2\leq k\leq n-1$, *we have*

24 $$ a^{(k+1)}\prec a^{(k)}. $$

### Proof

It is clear that (24) holds for any $n\geq 3$, $k=n-1$ and for $n= 3$, $k=1$.

Next, let $n\geq 4$ and $2\leq k\leq n-2$.

For any $1\leq m\leq n-k$ and $-1\leq r\leq k-2$, let $\sum_{i=0}^{-1}a ^{(k)}_{n-i}=0$, and let

$$ S_{m+r+1}^{(k)}= \sum_{i=1}^{m}a^{(k)}_{i}+ \sum_{i=0}^{r}a^{(k)}_{n-i}. $$

Next, we prove that

$$ S_{m+r+1}^{(k)}\geq S_{m+r+1}^{(k+1)} $$

in the following two cases:

(i) If $m=1$ and $-1\leq r\leq k-2$, then by Corollary 2.7(i) and Lemma 2.8 we get
  $$ S_{r+2}^{(k)}\geq S_{r+2}^{(k+1)}. $$
(ii) If $2\leq m\leq n-k$ and $-1\leq r\leq k-2$, then by Corollary 2.7(ii), Lemma 2.8, and Lemma 2.9 we get
  $$ S_{m+r+1}^{(k)}\geq S_{m+r+1}^{(k+1)}. $$
  Note that
  $$ S_{n}^{(k)}= S_{n}^{(k+1)}, $$
  so (24) holds.

 □

## Discussion

In the theory of majorizations, there are two key concepts, majorizing relations and Schur-convex functions. Majorizing relations are weaker ordered relations among vectors, and Shur-convex functions are an extension of classical convex functions. Combining these two objects is an effective method of constructing inequalities.

In the theory of majorization, there are two important and fundamental objects, establishing majorizing relations among vectors and finding various Schur-convex functions. Majorizing relations deeply characterize intrinsic connections among vectors, and combining a new majorizing relation with suitable Schur-convex functions can lead to various interesting inequalities; see [3–13].
